# A generalized Dunkl type modifications of Phillips operators

**DOI:** 10.1186/s13660-018-1909-2

**Published:** 2018-11-22

**Authors:** M. Nasiruzzaman, Nadeem Rao

**Affiliations:** 1 Department of Computer Science & Engineering (SEST), Jamia Hamdard, New Delhi, India; 2 Department of Civil Engineering, Jamia Millia Islamia University, New Delhi, India; 3 Department of Mathematics, Jamia Millia Islamia University, New Delhi, India

**Keywords:** 41A25, 41A36, 33C45, Szász operator, Generating functions, Dunkl analogue, Generalization of exponential function, Korovkin type theorem, Modulus of continuity, Order of convergence

## Abstract

The main purpose of this present article is to discuss the convergence of Lebesgue measurable functions by providing a Dunkl generalization of Szász type operators known as Phillips operators. To achieve the results of a better way of uniform convergence of the Phillips operators, we study qualitative results in a Korovkin and weighted Korovkin space.

## Introduction and preliminaries

In 1912 S.N. Bernstein [1] constructed positive linear operators for continuous functions defined on the interval $[0,1]$. The Bernstein operators preserve a simpler, more elegant, and constructive way that proves the first Weierstrass approximation theorem for the case $[0,1]$. In 1950 O. Szász [2] gave an extension to operators and constructed positive linear operators defined on $[0,\infty)$ for continuous functions defined on $[0,\infty)$. Presently working at pathways of the approximation process, several authors have obtained a Dunkl type generalization of Szász operators. This type of Dunkl generalization is a very recent work and it plays a crucial role in approximation theory. Firstly, the Dunkl type generalization was obtained by Sucu [3] who proposed an exponential generalization of the function given by [4]. They improved the Szász operators for a continuous function *f* defined on $[0,\infty)$, denoted as $f\in C[0,\infty)$ in which $x \geq0$, $\upsilon\ge0$, $n \in\mathbb{N}$, and constructed the following Dunkl type operators:

1.1 $$ \mathcal{S}_{n}^{\ast}(f;x):= \frac{1}{e_{\upsilon}(nx)} \sum_{r=0}^{\infty}\frac{(nx)^{r}}{\gamma_{\upsilon}(r)} f \biggl( \frac{r+2\upsilon\theta_{r}}{n} \biggr), $$

where

1.2 $$\begin{aligned}& e_{\upsilon}(x)= \sum_{k=0}^{\infty}\frac{x^{k}}{\gamma_{\upsilon}(k)}, \end{aligned}$$

1.3 $$\begin{aligned}& \gamma_{\upsilon}(2r)= \frac{2^{2r}r!\varGamma (r+\upsilon+\frac {1}{2} )}{ \varGamma (\upsilon+\frac{1}{2} )},\qquad \gamma_{\upsilon}(2r+1)= \frac{2^{2r+1}r!\varGamma (r+\upsilon+\frac {3}{2} )}{\varGamma (\upsilon+\frac{1}{2} )}. \end{aligned}$$

This type of generalization by exponential function was introduced, and it is a generalization of Hermite type polynomials, expressed in a form of the confluent hypergeometric function *Φ* (see [4]). For $r=0,1,2,\ldots$ , a recursion for $\gamma_{\upsilon}$

1.4 $$\begin{aligned}& \frac{\gamma_{\upsilon}(r+1)}{(r+1+2\upsilon\theta_{r+1})}=\gamma _{\upsilon}(r), \\& \theta_{r}=\textstyle\begin{cases} 0 & \text{if }r = 2n, n \in\mathbb{N}, \\ 1 & \text{if }r=2n+1, n\in\mathbb{N}.\end{cases}\displaystyle \end{aligned}$$

In the last quarter of the twentieth century, the quantum calculus, known as *q*-calculus, was studied by Jackson, Euler, and Jakobi (see [5, 6]). The application of *q*-calculus has most significance and efficiency in the field of sciences such as mathematics, physics, and chemistry which provide energetic ways to researchers. In recent times, the Dunkl type generalization of exponential functions attracted considerable attention to *q*-calculus and has been attractive to mathematicians. The Dunkl type generalizations of Szász operators have given an improvement to rise the *q*-calculus in approximation theory. For more details, we mention here some recent papers of Dunkl type generalization (see [7–13]). Moreover, in the recent years in the field of approximation theory the $(p,q)$-generalization of Bernstein operators was obtained by Mursaleen et al. [14], and then various operators have been generalized in $(p,q)$-analogue by different authors (see [15–23]).

The Dunkl type generalizations is a very recent crucial work of Szász operators to the approximation processes. Our work is to study and find the uniform approximation properties by Dunkl type generalizations to the Phillips operators [24]. The main ideas of our research methodologies include the estimations of degrees of Phillips approximating operators by using the properties of the modulus of continuity, Lipschitz functions, Peetre’s *K*-functional, and second order modulus of continuity. We have used a technique developed in [2, 3] and studied several uniform approximation properties of the Phillips operators by Dunkl generalizations; moreover, see also some of the recent papers [25–29]. In the present article the approximation obtained by these operators designed by Dunkl type provides a better generalization depending on *υ* and an educational platform to the researcher.

## Construction of operators and estimation of moments

For $\theta_{r}$ defined in (1.4) and each $f\in C_{\zeta}(\mathbb {R}^{+})=\{f\in C[0,\infty): f(t)=O(t^{\zeta})\}$ as $t\to\infty$, $x\in[0,\infty)$, $\zeta>n$, $n \in\mathbb{N}$, $\upsilon\geq0$, we define

2.1 $$ \mathcal{P}_{n,\upsilon}^{\ast}(f;x)= \frac{n^{2}}{e_{\upsilon }(nx)}\sum_{r=0}^{\infty} \frac{(nx)^{r}}{\gamma_{\upsilon}(k)} \int _{0}^{\infty}\frac{e^{-nt}n^{k+2\upsilon\theta_{r} -1}t^{r+2\upsilon \theta_{r} }}{\gamma_{\upsilon}(r)}f(t)\,\mathrm{d}t. $$

### Lemma 2.1

*Let* $e_{\ell}=t^{\ell-1}$ *for* $\ell=1,2,3,4,5$. *Then the operators* $\mathcal{P}_{n}^{\ast}( \cdot; \cdot)$ *defined by* (2.1) *satisfy the following identities*:

$$\begin{aligned}& (1)\quad\mathcal{P}_{n,\upsilon}^{\ast}(e_{1};x)=1, \\& (2) \quad\mathcal{P}_{n,\upsilon}^{\ast}(e_{2};x) = x+ \frac{1}{n}, \\& (3) \quad\mathcal{P}_{n,\upsilon}^{\ast}(e_{3};x) = \frac{2}{n^{2}} + \frac{2}{n} \biggl(2 + \upsilon\frac{e_{\upsilon}(-nx)}{e_{\upsilon}(nx)} \biggr)x+x^{2}, \\& (4)\quad\mathcal{P}_{n,\upsilon}^{\ast}(e_{4};x) = \frac{6}{n^{3}} + \frac{2}{n^{2}} \biggl(9 + 2\upsilon+ 8\upsilon \frac {e_{\upsilon}(-nx)}{e_{\upsilon}(nx)} \biggr)x+\frac{1}{n} \biggl(9 -2\upsilon \frac{e_{\upsilon}(-nx)}{e_{\upsilon}(nx)} \biggr)x^{2}+x^{3}, \\& (5)\quad\mathcal{P}_{n,\upsilon}^{\ast}(e_{5};x) = \frac{24}{n^{4}} + \frac{2}{n^{3}} \biggl(63 + 26\upsilon^{2}+ 2 \upsilon \bigl(29+2\upsilon^{2} \bigr) \frac{e_{\upsilon}(-nx)}{e_{\upsilon}(nx)} \biggr)x \\& \phantom{(5)\quad\mathcal{P}_{n,\upsilon}^{\ast}(e_{5};x) =}{}+\frac{4}{n^{2}} \biggl(18+\upsilon^{2} -7\upsilon \frac{e_{\upsilon}(-nx)}{e_{\upsilon}(nx)} \biggr)x^{2}+\frac{4}{n} \biggl(4+\upsilon \frac {e_{\upsilon}(-nx)}{e_{\upsilon}(nx)} \biggr)x^{3}+x^{4}. \end{aligned}$$

### Proof

Take $f(t)=e_{1}$, then

$$\begin{aligned} \mathcal{P}_{n,\upsilon}^{\ast}(e_{1};x)={}& \frac {n^{2}}{e_{\upsilon}(nx)}\sum_{r=0}^{\infty} \frac{(nx)^{r}}{\gamma _{\upsilon}(r)} \int_{0}^{\infty}\frac{e^{-nt}n^{r+2\upsilon\theta_{r} -1}t^{r+2\upsilon\theta_{r} }}{\gamma_{\upsilon}(r)}\,\mathrm{d}t \\ ={}&\frac{1}{e_{\upsilon}(nx)}\sum_{r=0}^{\infty} \frac{(nx)^{r}}{\gamma _{\upsilon}(r)} \int_{0}^{\infty}\frac{e^{-t}t^{r+2\upsilon\theta_{r} }}{\gamma_{\upsilon}(r)}\,\mathrm{d}t = \frac{1}{e_{\upsilon}(nx)}\sum_{r=0}^{\infty} \frac{(nx)^{r}}{\gamma _{\upsilon}(r)}\frac{\varGamma(r+2\upsilon\theta_{r}+1 )}{\gamma_{\upsilon }(r)} \\ ={}&\frac{1}{e_{\upsilon}(nx)}\sum_{r=0}^{\infty} \frac{(nx)^{r}}{\gamma _{\upsilon}(r)} =1.\end{aligned} $$

Take $f(t)=e_{2}$, then

$$\begin{aligned} \mathcal{P}_{n,\upsilon}^{\ast}(e_{2};x)&=\frac{n^{2}}{e_{\upsilon }(nx)} \sum_{r=0}^{\infty}\frac{(nx)^{r}}{\gamma_{\upsilon}(r)} \int _{0}^{\infty}\frac{e^{-nt}n^{r+2\upsilon\theta_{r} -1}t^{+}2\upsilon\theta _{r}+1 }{\gamma_{\upsilon}(r)}\,\mathrm{d}t \\ &=\frac{1}{ne_{\upsilon}(nx)}\sum_{r=0}^{\infty} \frac{(nx)^{r}}{\gamma _{\upsilon}(r)} \int_{0}^{\infty}\frac{e^{-t}t^{r+2\upsilon\theta_{r}+1 }}{\gamma_{\upsilon}(r)}\,\mathrm{d}t \\ &=\frac{1}{ne_{\upsilon}(nx)}\sum_{r=0}^{\infty} \frac{(nx)^{r}}{\gamma _{\upsilon}(r)}\frac{\varGamma(r+2\upsilon\theta_{r}+2 )}{\gamma_{\upsilon}(r)} =\frac{1}{ne_{\upsilon}(nx)}\sum _{r=0}^{\infty}\frac{(nx)^{r}}{\gamma _{\upsilon}(r)}(r+2\upsilon \theta_{r}+1 ) \\ &=\frac{1}{ne_{\upsilon}(nx)}\sum_{r=0}^{\infty} \frac{(nx)^{r}}{\gamma _{\upsilon}(r)} +\frac{1}{ne_{\upsilon}(nx)}\sum_{r=0}^{\infty} \frac{(nx)^{r}}{\gamma _{\upsilon}(r)}(r+2\upsilon\theta_{r} ) \\ &= x+\frac{1}{n}.\end{aligned} $$

Take $f(t)=e_{3}$, then

$$\begin{aligned} \mathcal{P}_{n,\upsilon}^{\ast}(e_{3};x) =& \frac{n^{2}}{e_{\upsilon}(nx)}\sum_{r=0}^{\infty} \frac{(nx)^{r}}{\gamma_{\upsilon}(r)} \int_{0}^{\infty }\frac{e^{-nt}n^{r+2\upsilon\theta_{r} -1}t^{r+2\upsilon\theta_{r}+2 }}{\gamma_{\upsilon}(r)}\,\mathrm{d}t \\ =&\frac{1}{n^{2}e_{\upsilon}(nx)}\sum_{r=0}^{\infty} \frac{(nx)^{r}}{\gamma _{\upsilon}(r)} \int_{0}^{\infty}\frac{e^{-t}t^{r+2\upsilon\theta_{r}+2 }}{\gamma_{\upsilon}(r)}\,\mathrm{d}t\\ =& \frac{1}{n^{2}e_{\upsilon}(nx)}\sum_{r=0}^{\infty} \frac{(nx)^{r}}{\gamma _{\upsilon}(r)}\frac{\varGamma(r+2\upsilon\theta_{r}+3 )}{\gamma_{\upsilon }(r)} \\ =&\frac{1}{n^{2}e_{\upsilon}(nx)}\sum_{r=0}^{\infty} \frac{(nx)^{r}}{\gamma _{\upsilon}(r)}(r+2\upsilon\theta_{r}+2 ) (r+2\upsilon \theta_{r}+1 ) \\ =&\frac{1}{n^{2}e_{\upsilon}(nx)}\sum_{r=0}^{\infty} \frac{(nx)^{r}}{\gamma _{\upsilon}(r)} \bigl((r+2\upsilon\theta_{r} )^{2}+3(r+2 \upsilon\theta_{r} )+2 \bigr) \\ =&\frac{1}{n^{2}e_{\upsilon}(nx)}\sum_{r=0}^{\infty} \frac{(nx)^{r}}{\gamma _{\upsilon}(r)}(r+2\upsilon\theta_{r} )^{2} \\ & {}+\frac{3}{n^{2}e_{\upsilon}(nx)}\sum_{r=0}^{\infty} \frac{(nx)^{r}}{\gamma _{\upsilon}(r)}(r+2\upsilon\theta_{r} ) +\frac{2}{n^{2}e_{\upsilon}(nx)}\sum _{r=0}^{\infty}\frac{(nx)^{r}}{\gamma _{\upsilon}(r)} \\ =&\frac{2}{n^{2}} + \frac{2}{n} \biggl(2 + \upsilon\frac{e_{\upsilon}(-nx)}{e_{\upsilon}(nx)} \biggr)x+x^{2}. \end{aligned}$$

By the simple calculation, other results can easily be obtained. □

### Lemma 2.2

*For* $e_{\ell}=t^{\ell-1}$, $\ell=1,2,3,4,5$, *suppose* $\eta_{j} =(e_{2}-x)^{j}$ *for* $j=1,2,3,4$. *The operators* $\mathcal{P}_{n,\upsilon}^{\ast}( \cdot; \cdot)$ *defined by* (2.1) *satisfy the following identities*:

$$\begin{gathered} 1^{\circ}\quad \mathcal{P}_{n,\upsilon}^{\ast}( \eta_{1};x) =\frac{1}{n}, \\ 2^{\circ}\quad \mathcal{P}_{n,\upsilon}^{\ast}( \eta_{2};x) = \frac {2}{n^{2}} + \frac{2}{n} \biggl(1 + \upsilon\frac{e_{\upsilon}(-nx)}{e_{\upsilon}(nx)} \biggr)x, \\ 3^{\circ}\quad \mathcal{P}_{n,\upsilon}^{\ast}( \eta_{4};x) = \frac {24}{n^{3}} + \frac{2}{n^{3}} \biggl(51 + 26 \upsilon^{2}+2\upsilon \bigl(29+2\upsilon ^{2} \bigr) \frac{e_{\upsilon}(-nx)}{e_{\upsilon}(nx)} \biggr)x \\ \phantom{3^{\circ}\quad \mathcal{P}_{n,\upsilon}^{\ast}( \eta_{4};x) =}{}+ \frac{4}{n^{2}} \biggl(3 -4\upsilon+\upsilon^{2}-23\upsilon \frac {e_{\upsilon}(-nx)}{e_{\upsilon}(nx)} \biggr)x^{2}+\frac{24}{n}\upsilon \frac {e_{\upsilon}(-nx)}{e_{\upsilon}(nx)}x^{3}.\end{gathered} $$

### Proof

We use the linearity property $\mathcal{P}_{n,\upsilon}^{\ast}(\eta_{1};x) =\mathcal{P}_{n,\upsilon}^{\ast}(e_{2};x)-x\mathcal{P}_{n,\upsilon}^{\ast}(e_{1};x)$, $\mathcal{P}_{n,\upsilon}^{\ast}(\eta_{2};x) =\mathcal{P}_{n,\upsilon}^{\ast}(e_{3};x)-2x\mathcal{P}_{n,\upsilon}^{\ast}(e_{2};x)+x^{2}\mathcal {P}_{n,\upsilon}^{\ast}(e_{1};x)$ and $\mathcal{P}_{n,\upsilon}^{\ast}(\eta_{4};x)=\mathcal{P}_{n,\upsilon}^{\ast}(e_{5};x)-4x\mathcal{P}_{n,\upsilon}^{\ast}(e_{4};x)+ 6x^{2}\mathcal{P}_{n,\upsilon}^{\ast}(e_{3};x)-4x^{3}\mathcal{P}_{n,\upsilon }^{\ast}(e_{2};x)+x^{4}\mathcal{P}_{n,\upsilon}^{\ast}(e_{1};x)$. □

## Convergence in Korovkin and weighted Korovkin space

The Korovkin’ type approximation theory has many useful connections with the classical approximation theory as well as with other branches of mathematics. In the present section the results related to uniform convergence of the operators defined by (2.1) via the well-known Korovkin’ and weighted Korovkin’ type theorems are obtained.

In the present article, the set of all functions which are bounded and continuous on $[0,\infty)=\mathbb{R^{+}}$, denoted by $C_{B}[0,\infty )$ and $C[0,\infty)$, denotes the set of all continuous functions in $C_{B}[0,\infty)$. Also the linear normed space with the supremum norm is defined as follows:

$$ \Vert f \Vert _{C_{B[0,\infty)}}=\sup_{x\geq0} \bigl\vert f(x) \bigr\vert . $$

Let

$$ E:= \bigl\{ f:x\in{}[0,\infty) \bigr\} $$

for which the function $\frac{f(x)}{1+x^{2}}$ is uniformly convergent as it approaches ∞.

### Theorem 3.1

*Let the function* $f\in C[0,\infty)\cap E$ *and the operators* $\mathcal{P}_{n,\upsilon}^{\ast}( \cdot; \cdot)$ *be defined by* (2.1). *Then*

$$ \lim_{n\rightarrow\infty}\mathcal{P}_{n,\upsilon}^{\ast}(f;x) = f(x) $$

*is uniform on each compact subset of* $[0,\infty)$.

### Proof

To prove the uniformity of the operators $\mathcal{P}_{n,\upsilon}^{\ast}$, the well-known Korovkin theorem is used. So, for $\ell=1,2,3$, as *n* approaches ∞, we prove the three conditions. Therefore, $\lim_{n\rightarrow\infty}\mathcal{P}_{n,\upsilon}^{\ast}((e_{\ell};x)\rightarrow x^{\ell}$ is uniformly convergent on $[0,\infty)$. Clearly, as $(n\rightarrow \infty)$, then $\frac{1}{n}\rightarrow0$. Hence we have

$$ \lim_{n \to\infty}\mathcal{P}_{n,\upsilon}^{\ast}(t;x)= x,\quad\quad \lim_{n \to \infty}\mathcal{P}_{n,\upsilon}^{\ast} \bigl(t^{2};x \bigr)= x^{2}, $$

which completes the proof. □

Take $\sigma(x)=1+x^{2}$ is a weight function and the functions $f\in C[0,\infty)$ are defined in weighted spaces for which

$$\begin{aligned}& P_{\sigma}(x) {\big|}_{x\in[0,\infty)} = \bigl\{ f: \bigl\vert f(x) \bigr\vert \leq M_{f}\sigma (x) \bigr\} , \\& Q_{\sigma}(x) {\big|}_{x\in[0,\infty)} = \bigl\{ f:f\in P_{\sigma }(x) \cap C[0,\infty) \bigr\} , \\& Q_{\sigma}^{m}(x) {\big|}_{x\in[0,\infty)} = \biggl\{ f:f\in Q_{\sigma }(x) \mbox{ and } \lim_{x\rightarrow\infty} \frac{f(x)}{\sigma(x)}=m \biggr\} , \end{aligned}$$

where $M_{f}$ depends only on *f* and is a constant. It should be noted that, for $x \in[0,\infty)$, $Q_{\sigma}(x)$ is a normed space defined with the norm of $\Vert f\Vert_{\sigma}=\sup_{x\in[0,\infty)}\frac{\vert f(x)\vert}{\sigma(x)}$.

### Theorem 3.2

*Let* $\mathcal{P}_{n,\upsilon}^{\ast}( \cdot; \cdot)$ *be the operators defined by* (2.1). *Then*, *for* $f \in Q_{\sigma}^{m}(x) {|}_{x\in[0,\infty)}$, *we have*

$$ \lim_{n\to\infty} \bigl\Vert \mathcal{P}_{n,\upsilon}^{\ast}(f;x)-f \bigr\Vert _{\sigma}=0. $$

### Proof

Suppose $f(t) \in C^{m}_{\sigma}(\mathbb{R}^{+})$, and if we take $f(t)=t^{\tau}$, then by the Korovkin theorem if it satisfies $\mathcal{P}_{n,\upsilon}^{\ast}(t^{\tau};x)\rightarrow x^{\tau}$, for $\tau =0,1,2$ uniformly, whenever $n \to\infty$, then from the case when $\tau=0$, by applying Lemma 2.1, since $\mathcal {P}_{n,\upsilon}^{\ast}(1;x)=1$, we have

3.1 $$ \big\| \mathcal{P}_{n,\upsilon}^{\ast}(1;x ) -1\big\| _{\sigma} =0. $$

For $\tau=1$, we have

$$\big\| \mathcal{P}_{n,\upsilon}^{\ast}(t;x ) -x \big\| _{\sigma} = \sup _{x \in[0,\infty)}\frac{ \vert \mathcal{P}_{n,\upsilon }^{\ast}(t;x)-x \vert }{1+x^{2}}=\frac{1}{n}\sup _{x \in[0,\infty)}\frac{1}{1+x^{2}}. $$

As $n \to\infty$, then

3.2 $$ \big\| \mathcal{P}_{n,\upsilon}^{\ast}(t;x ) -x\big\| _{\sigma} =0. $$

In a similar way, for $\tau=2$,

3.3 $$\begin{aligned}& \begin{aligned} \big\| \mathcal{P}_{n,\upsilon}^{\ast}\bigl(t^{2};x \bigr) -x^{2} \big\Vert _{\sigma} &= \sup_{x \in[0,\infty)}\frac{ \vert \mathcal{P}_{n,\upsilon }^{\ast}(t^{2};x)-x^{2} \vert }{1+x^{2}} \\ & = \frac{2}{n} \biggl(2 + \upsilon\frac{e_{\upsilon}(-nx)}{e_{\upsilon}(nx)} \biggr)\sup _{x \in[0,\infty)}\frac {x}{1+x^{2}}+\frac{2}{n^{2}}\sup _{x \in[0,\infty)}\frac{1}{1+x^{2}},\end{aligned} \\ & \big\Vert \mathcal{P}_{n,\upsilon}^{\ast}\bigl(t^{2};x \bigr) -x^{2}\big\Vert _{\sigma} =0 \quad (\text{whenever } n \to\infty), \end{aligned}$$

which completes the proof. □

## Order of approximation

Let $H=\{f { |} f \in\tilde{C}[0,\infty)\}$, whenever $\tilde {C}[0,\infty)$ is the space of uniformly continuous functions on $[0,\infty)$ and $\tilde{\omega}(f;\tilde{\delta} )$ is the modulus of continuity of the function $f \in\tilde{C}[0,\infty)$ which are enabled to give a maximum oscillation of *f* for $\tilde{\delta} >0$. One has

4.1 $$ \tilde{\omega} (f;\tilde{\delta} )=\sup_{ \vert x_{1}-x_{2} \vert \leq\tilde {\delta} } \bigl\vert f(x_{1})-f(x_{2}) \bigr\vert ;\quad x_{1},x_{2} \in{}[0,\infty). $$

It should be noted that, for $f\in\tilde{C}[0,\infty)$, $\tilde{\delta} >0$, we have $\lim_{\tilde {\delta} \rightarrow0+}\tilde{\omega} (f;\tilde{\delta} )=0$,

4.2 $$ \bigl\vert f(x_{1})-f(x_{2}) \bigr\vert \leq \biggl( \frac{ \vert x_{1}-x_{2} \vert }{\tilde {\delta} }+1 \biggr) \tilde{\omega} (f;\tilde{\delta} ). $$

### Theorem 4.1

*Let the function* $f \in H$, $x\in[0,\infty)$ *and the operators* $\mathcal {P}_{n,\upsilon}^{\ast}( \cdot; \cdot)$ *be defined by* (2.1). *Then*

$$ \bigl\vert \mathcal{P}_{n,\upsilon}^{\ast}(f;x)-f(x) \bigr\vert \leq \biggl\{ 1+\sqrt{\frac{2}{n} + 2 \biggl(1 + \upsilon\frac{e_{\upsilon}(-nx)}{e_{\upsilon}(nx)} \biggr)x} \biggr\} \tilde{\omega} (f;\tilde {\delta} _{n} ). $$

### Proof

We used the Cauchy–Schwarz inequality and the results defined by (4.1), (4.2). Hence

$$\begin{gathered} \bigl\vert \mathcal{P}_{n,\upsilon}^{\ast}(f;x)-f(x) \bigr\vert \\ \quad\leq \frac{n^{2}}{e_{\upsilon}(nx)}\sum_{r=0}^{\infty} \frac {(nx)^{r}}{\gamma_{\upsilon}(r)} \int_{0}^{\infty}\frac {e^{-nt}n^{r+2\upsilon\theta_{r} -1}t^{r+2\upsilon\theta_{r} }}{\gamma _{\upsilon}(r)} \bigl\vert f(t)-f(x) \bigr\vert \,\mathrm{d}t \\ \quad\leq\frac{n^{2}}{e_{\upsilon}(nx)}\sum_{r=0}^{\infty} \frac {(nx)^{r}}{\gamma_{\upsilon}(r)} \int_{0}^{\infty}\frac {e^{-nt}n^{r+2\upsilon\theta_{r} -1}t^{r+2\upsilon\theta_{r} }}{\gamma _{\upsilon}(r)} \biggl( 1+ \frac{1}{\tilde{\delta} } \vert t-x \vert \biggr) \tilde{\omega} (f;\tilde{\delta} )\, \mathrm{d}t \\ \quad= { \Biggl\{ } 1+\frac{1}{\tilde{\delta}} \Biggl(\frac{n^{2}}{e_{\upsilon }(nx)}\sum _{r=0}^{\infty}\frac{(nx)^{r}}{\gamma_{\upsilon}(r)} \int _{0}^{\infty}\frac{e^{-nt}n^{r+2\upsilon\theta_{r} -1}t^{r+2\upsilon \theta_{r} }}{\gamma_{\upsilon}(r)} \vert t-x \vert \,\mathrm{d}t \Biggr) { \Biggr\} } \tilde{\omega} (f;\tilde{\delta} ) \\ \quad\leq { \Biggl\{ } 1+\frac{1}{\tilde{\delta} } { \Biggl(} \frac {n^{2}}{e_{\upsilon}(nx)}\sum _{r=0}^{\infty}\frac{(nx)^{r}}{\gamma _{\upsilon}(r)} \int_{0}^{\infty}\frac{e^{-nt}n^{r+2\upsilon\theta_{r} -1}t^{r+2\upsilon\theta_{r} }}{\gamma_{\upsilon}(r)} (t-x)^{2} \,\mathrm {d}t { \Biggr)} ^{\frac{1}{2}} \bigl( \mathcal{P}_{n,\upsilon}^{\ast}(1;x) \bigr) ^{\frac{1}{2}} { \Biggr\} } \\ \qquad{}\times\tilde{\omega} (f;\tilde{\delta} ) \\ \quad= \tilde{\omega} (f;\tilde{\delta} )+\frac{1}{\tilde{\delta} } \bigl( \mathcal{P}_{n,\upsilon}^{\ast}(\eta_{2};x) \bigr) ^{\frac{1}{2}} \tilde{\omega} (f;\tilde{\delta} ).\end{gathered} $$

Choose $\tilde{\delta} =\sqrt{\frac{1}{n}}=\tilde{\delta} _{n}$, then we get our result. □

### Corollary 4.2

*For* $f \in H$, $x\in[0,\infty)$ *and* $\tilde{\delta} _{n}=\mathcal {P}_{n,\upsilon}^{\ast}(\eta_{2};x)$,

$$ \bigl\vert \mathcal{P}_{n,\upsilon}^{\ast}(f;x)-f(x) \bigr\vert \leq2 \tilde{\omega} (f;\tilde{\delta} _{n} ). $$

## Rate of convergence

In the present section we use the usual class of Lipschitz functions and obtain the rate of convergence of the sequence of positive linear operators $\mathcal{P}_{n,\upsilon}^{\ast}(f;x) $ (2.1) for which the operators uniformly converge to the continuous function *f* on $[0,\infty)$.

For $\mathcal{C}>0$, $0<\nu\leq1$, and for the continuous functions *f* on $[0,\infty)$, the class of Lipschitz functions $\operatorname{Lip}_{\mathcal{C},\nu }(f)$ is

5.1 $$ \operatorname{Lip}_{\mathcal{C},\nu}(f)= \bigl\{ f: \bigl\vert f(\varsigma_{1})-f( \varsigma _{2}) \bigr\vert \leq\mathcal{C} \vert \varsigma_{1}- \varsigma_{2} \vert ^{\nu}; \bigl( \varsigma_{1}, \varsigma _{2}\in{}[ 0,\infty)\bigr) \bigr\} . $$

### Theorem 5.1

*Let* $f\in \operatorname{Lip}_{\mathcal{C},\nu}$, *for* $\mathcal{C}>0$, $0<\nu\leq1$. *Suppose that* $\mathcal{P}_{n,\upsilon}^{\ast}( \cdot; \cdot)$ *are the positive linear operators defined in* (2.1). *Then*

$$ \bigl\vert \mathcal{P}_{n,\upsilon}^{\ast}(f;x)-f(x) \bigr\vert \leq\mathcal{C} \biggl(\frac{2}{n^{2}} + \frac{2}{n} \biggl(1 + \upsilon \frac{e_{\upsilon}(-nx)}{e_{\upsilon}(nx)} \biggr)x \biggr)^{\frac{\nu}{2}}. $$

### Proof

By applying the Hölder inequality and (5.1), we get

$$\begin{aligned} \bigl\vert \mathcal{P}_{n,\upsilon}^{\ast}(f;x)-f(x) \bigr\vert & \leq \bigl\vert \mathcal {P}_{n,\upsilon}^{\ast}\bigl(f(t)-f(x);x \bigr) \bigr\vert \\ &\leq\mathcal{P}_{n,\upsilon}^{\ast}\bigl( \bigl\vert f(t)-f(x) \bigr\vert ;x \bigr) \\ &\leq \mathcal{C} \mathcal{P}_{n,\upsilon}^{\ast}\bigl( \vert t-x \vert ^{\nu};x \bigr) .\end{aligned} $$

Therefore,

$$\begin{gathered} \bigl\vert \mathcal{P}_{n,\upsilon}^{\ast}(f;x)-f(x) \bigr\vert \\ \quad\leq \mathcal{C} \frac{n^{2}}{e_{\upsilon}(nx)}\sum_{r=0}^{\infty } \frac{(nx)^{r}}{\gamma_{\upsilon}(r)} \int_{0}^{\infty}\frac {e^{-nt}n^{r+2\upsilon\theta_{r} -1}t^{r+2\upsilon\theta_{r} }}{\gamma _{\upsilon}(r)} \vert t - x \vert \,\mathrm{d}t \\ \quad\leq \mathcal{C}\frac{n^{2}}{e_{\upsilon}(nx)}\sum_{r=0}^{\infty} \biggl(\frac{(nx)^{r}}{\gamma_{\upsilon}(r)} \biggr)^{\frac{2-\nu}{2}} \biggl(\frac{(nx)^{r}}{\gamma_{\upsilon}(r)} \biggr)^{\frac{\nu}{2}} \int_{0}^{\infty}\frac{e^{-nt}n^{r+2\upsilon\theta_{r} -1}t^{r+2\upsilon\theta_{r} }}{\gamma_{\upsilon}(r)} \vert t - x \vert \,\mathrm{d}t \\ \quad\leq \mathcal{C} \Biggl(\frac{n^{2}}{e_{\upsilon}(nx)}\sum_{r=0}^{\infty} \frac{(nx)^{r}}{\gamma_{\upsilon}(r)} \int_{0}^{\infty }\frac{e^{-nt}n^{r+2\upsilon\theta_{r} -1}t^{r+2\upsilon\theta_{r} }}{\gamma_{\upsilon}(r)} \,\mathrm{d}t \Biggr)^{\frac{2-\nu}{2}} \\ \qquad{}\times \Biggl(\frac{n^{2}}{e_{\upsilon}(nx)}\sum_{r=0}^{\infty} \frac {(nx)^{r}}{\gamma_{\upsilon}(r)} \int_{0}^{\infty}\frac {e^{-nt}n^{r+2\upsilon\theta_{r} -1}t^{r+2\upsilon\theta_{r} }}{\gamma _{\upsilon}(r)} \vert t - x \vert ^{2} \,\mathrm{d}t \Biggr)^{\frac{\nu}{2}} \\ \quad= \mathcal{C} \bigl(\mathcal{P}_{n,\upsilon}^{\ast}(t-x)^{2};x \bigr)^{\frac{\nu}{2}}=\mathcal{C} \bigl(\mathcal{P}_{n,\upsilon}^{\ast}( \eta _{2};x) \bigr)^{\frac{\nu}{2}},\end{gathered} $$

which completes the proof. □

The space of all the functions that are continuous and bounded on $\mathbb{R}^{+}=[0,\infty)$ is denoted by $C_{B}(\mathbb{R}^{+})$. Hence one has

5.2 $$ C_{B}^{2} \bigl(\mathbb{R}^{+} \bigr)= \bigl\{ \psi \in C_{B} \bigl(\mathbb{R}^{+} \bigr): \psi^{\prime }, \psi^{\prime \prime}\in C_{B} \bigl( \mathbb{R}^{+} \bigr) \bigr\} , $$

with the norm defined on $C_{B}^{2}(\mathbb{R}^{+})$, written as

5.3 $$ \Vert \psi \Vert _{C_{B}^{2}(\mathbb{R}^{+})}= \bigl\Vert \psi ^{\prime\prime} \bigr\Vert _{C_{B}(\mathbb{R}^{+})}+ \bigl\Vert \psi ^{\prime} \bigr\Vert _{C_{B}(\mathbb{R}^{+})}+ \Vert \psi \Vert _{C_{B}(\mathbb{R}^{+})}, $$

where the norm is defined on $C_{B}[0,\infty)$,

5.4 $$ \Vert \psi \Vert _{C_{B}(\mathbb{R}^{+})}=\sup_{x\in[0,\infty )} \bigl\vert \psi(x) \bigr\vert . $$

### Theorem 5.2

*Let the operators* $\mathcal{P}_{n,\upsilon}^{\ast}( \cdot; \cdot)$ *be defined in* (2.1). *Then*, *for every* $\psi\in C_{B}^{2}(\mathbb {R}^{+})$ *defined by* (5.2), *we have*

$$ \bigl\vert \mathcal{P}_{n,\upsilon}^{\ast}(\psi;x)-\psi(x) \bigr\vert \leq ( \varTheta_{n} + \varLambda_{n,x} ) \Vert \psi \Vert _{C_{B}^{2}(\mathbb{R}^{+})}, $$

*where* $\varTheta_{n}=\frac{1}{n}+\frac{1}{n^{2}}$ *and* $\varLambda_{n,x}= \frac {1}{n} (1 + \upsilon\frac{e_{\upsilon}(-nx)}{e_{\upsilon}(nx)} )x$.

### Proof

Let $\psi\in C_{B}^{2}(\mathbb{R}^{+})$. From the expansion of Taylor series, the generalized mean value theorem, we have

$$ \psi(t)=\psi(x)+(t-x)\psi^{\prime}(x)+\frac{(t-x)^{2}}{2} \psi^{\prime \prime}(\varphi)\frac{(t-x)^{2}}{2},\quad \varphi\in(x,t). $$

A small calculation leads to linearity on $\mathcal{P}_{n,\upsilon}^{\ast}$, we have

$$ \mathcal{P}_{n,\upsilon}^{\ast}(\psi;x)-\psi(x)=\psi^{\prime}(x) \mathcal {P}_{n,\upsilon}^{\ast}\bigl( (t-x);x \bigr) + \frac{\psi^{\prime\prime}(\varphi)}{2}\mathcal{P}_{n,\upsilon}^{\ast}\bigl( (t-x)^{2};x \bigr) , $$

which implies that

$$\begin{gathered} \bigl\vert \mathcal{P}_{n,\upsilon}^{\ast}(\psi;x)-\psi(x) \bigr\vert \leq \biggl(\frac{1}{n} \biggr) \bigl\Vert \psi^{\prime} \bigr\Vert _{C_{B}(\mathbb{R}^{+})} + { \biggl\{ } \frac{2}{n^{2}} + \frac{2}{n} \biggl(1 + \upsilon \frac {e_{\upsilon}(-nx)}{e_{\upsilon}(nx)} \biggr)x { \biggr\} } \frac{ \Vert \psi ^{\prime\prime } \Vert _{C_{B}(\mathbb{R}^{+})}}{2}.\end{gathered} $$

From (5.3) we have $\Vert\psi^{\prime}\Vert _{C_{B}[0,\infty)}\leq\Vert\psi\Vert_{C_{B}^{2}[0,\infty )}$ and $\Vert\psi^{\prime \prime}\Vert _{C_{B}(\mathbb{R}^{+})}\leq\Vert\psi\Vert_{C_{B}^{2}(\mathbb{R}^{+})}$.

$$\begin{gathered} \bigl\vert \mathcal{P}_{n,\upsilon}^{\ast}(\psi;x)-\psi(x) \bigr\vert \leq \biggl(\frac{1}{n} \biggr) \Vert \psi \Vert _{C_{B}^{2}(\mathbb{R}^{+})} + { \biggl\{ } \frac{2}{n^{2}} + \frac{2}{n} \biggl(1 + \upsilon \frac {e_{\upsilon}(-nx)}{e_{\upsilon}(nx)} \biggr)x { \biggr\} } \frac{ \Vert \psi \Vert _{C_{B}^{2}(\mathbb{R}^{+})}}{2}.\end{gathered} $$

 □

## Convergence properties of some direct theorem

A potential influences work to obtain a well-known functional known as Peetre’s *K*-functional, given by J. Peetre in 1968. The conflict of interest for *K*-functional to investigate the interpolation spaces between two Banach spaces and interactions to the real interpolation is based on *K*-functional.

This well-known functional property, which is known as *K*-functional, was defined by Peetre as follows:

6.1 $$ \mathcal{K}_{2}(f,\breve{\delta} )=\inf_{C_{B}^{2}(\mathbb{R}^{+})} \bigl\{ \bigl( \Vert f-\psi \Vert _{C_{B}([0,\infty))}+\breve{\delta} \Vert \psi \Vert _{C_{B}^{2}([0,\infty))} \bigr) :\psi\in C_{B}^{2} \bigl( \mathbb {R}^{+} \bigr) \bigr\} . $$

For any $\breve{\delta} >0$, there exists a positive constant $\mathcal {C}>0$ such that $\mathcal{K}_{2}(f,\breve{\delta} )\leq \mathcal{C}\omega_{2}(f,\breve{\delta} ^{\frac{1}{2}})$, where the second order modulus of continuity is given by

6.2 $$ \omega_{2} \bigl(f,\breve{\delta} ^{\frac{1}{2}} \bigr)=\sup _{0< h< \breve{\delta} ^{\frac{1}{2}}}\sup_{t \in[0,\infty)} \bigl\vert f(t+2h)-2f(t+h)+f(t) \bigr\vert . $$

### Theorem 6.1

*Let* $f\in C_{B}^{2}(\mathbb{R}^{+})$, $x\in[0,\infty)$, *and the operators* $\mathcal{P}_{n,\upsilon}^{\ast}( \cdot; \cdot)$ *be defined by* (2.1). *Then we have*

$$ \bigl\vert \mathcal{P}_{n,\upsilon}^{\ast}(f;x)-f(x) \bigr\vert \leq 2\mathcal{D} { \biggl\{ } \omega_{2} \biggl( f;\sqrt{ \frac{\varTheta_{n} + \varLambda_{n,x}}{2}} \biggr) + \min \biggl( 1,\frac{\varTheta_{n} + \varLambda_{n,x}}{2} \biggr) \Vert f \Vert _{C_{B}(\mathbb{R}^{+})} { \biggr\} }, $$

*where* $\omega_{2}(f;\breve{\delta} )$ *is defined in* (6.2) *and* $\mathcal{D}$ *is a nonnegative constant*.

### Proof

We use the results obtained in Theorem (5.2) and get

$$\begin{aligned} \bigl\vert \mathcal{P}_{n,\upsilon}^{\ast}(f;x)-f(x) \bigr\vert & \leq \bigl\vert \mathcal {P}_{n,\upsilon}^{\ast}(f-\psi;x) \bigr\vert + \bigl\vert \mathcal{P}_{n,\upsilon}^{\ast}( \psi;x)-\psi(x) \bigr\vert + \bigl\vert f(x)-\psi(x) \bigr\vert \\ &\leq 2 \Vert f-\psi \Vert _{C_{B}(\mathbb{R}^{+})}+ (\varTheta _{n} + \varLambda_{n,x} ) \Vert \psi \Vert _{C_{B}^{2}(\mathbb{R}^{+})} \\ &= 2 \biggl( \Vert f-\psi \Vert _{C_{B}(\mathbb{R}^{+})}+\frac{\varTheta_{n} + \varLambda_{n,x}}{2} \Vert \psi \Vert _{C_{B}^{2}(\mathbb{R}^{+})} \biggr).\end{aligned} $$

By taking infimum over all $\psi\in C_{B}^{2}(\mathbb{R}^{+})$ and using the results obtained by (6.1), we get

$$ \bigl\vert \mathcal{P}_{n,\upsilon}^{\ast}(f;x)-f(x) \bigr\vert = 2K_{2} \biggl( f;\frac {\varTheta_{n} + \varLambda_{n,x}}{2} \biggr). $$

Now, from the article [30] an absolute constant $\mathcal{D}>0$ exists, so we use here

$$ \mathcal{K}_{2}(f;\breve{\delta} )\leq\mathcal{D} \bigl\{ \min(1, \breve {\delta} ) \Vert f \Vert _{C_{B}(\mathbb{R}^{+})}+\omega_{2}(f; \sqrt{\breve {\delta} }) \bigr\} . $$

This completes the proof. □

Atakut and Ispir [31] introduced the weighted modulus of continuity and defined it as follows: for an arbitrary $f\in Q_{\sigma}^{m}(x)$,

6.3 $$ \bar{\varOmega} (f;\hat{\delta} )=\sup_{ \vert h \vert \leq\hat{\delta}, x\in {}[0,\infty) }\frac{ \vert f(x+h)-f(x) \vert }{(1+x^{2})(1+h^{2})}, $$

with the properties defined as

6.4 $$\begin{aligned}& \lim_{\hat{\delta} \rightarrow0}\bar{\varOmega} (f;\hat{\delta} )= 0, \end{aligned}$$

6.5 $$\begin{aligned}& \bigl\vert f(t)-f(x) \bigr\vert \leq2 \biggl( \frac{ \vert t-x \vert }{\hat{\delta} }+1 \biggr) \bigl(1+\hat{ \delta}^{2} \bigr) \bigl(1+x^{2} \bigr) \bigl((t-x)^{2}+1 \bigr)\bar{\varOmega} (f;\hat{\delta} ), \end{aligned}$$

where $f\in Q_{\sigma}^{m}(x)$ and $t,x\in{}[0,\infty)$.

### Theorem 6.2

*Let* $f \in Q^{m}_{\sigma}(x)$, $x\in[0,\infty)$, *then for the operators* $\mathcal {P}_{n,\upsilon}^{\ast}( \cdot; \cdot)$ *defined by* (2.1), *we have*

$$ \sup_{x \in[0,\infty)}\frac{ \vert \mathcal{P}_{n,\upsilon}^{\ast}(f;x)-f(x) \vert }{(1+x^{2})^{\frac{3}{2}}} \leq 2\mathcal{M}_{\upsilon}\bigl( 1+\mathfrak{W}_{\upsilon}(n) \bigr)\bar {\varOmega} \bigl( f;\sqrt{ \mathfrak{W}_{\upsilon}(n)} \bigr), $$

*where the constant* $\mathcal{M}_{\upsilon}$ *does not depend on* *n* *and* $\mathfrak{W}_{\upsilon}(n)=\max {\{}\frac{2}{n^{2}}, \frac {2}{n} (1 + \upsilon\frac{e_{\upsilon}(-nx)}{e_{\upsilon}(nx)} ) {\}}$.

### Proof

We prove it by using (6.3), (6.5), and the Cauchy–Schwarz inequality.

$$\begin{gathered} \bigl\vert \mathcal{P}_{n,\upsilon}^{\ast}(f;x)-f(x) \bigr\vert \\ \quad\leq\frac{n^{2}}{e_{\upsilon}(nx)}\sum_{r=0}^{\infty} \frac {(nx)^{r}}{\gamma_{\upsilon}(r)} \int_{0}^{\infty}\frac {e^{-nt}n^{r+2\upsilon\theta_{r} -1}t^{r+2\upsilon\theta_{r} }}{\gamma _{\upsilon}(r)} \bigl\vert f(t) - f(x) \bigr\vert \,\mathrm{d}t \\ \quad\leq2 \bigl(1+\hat{\delta} ^{2} \bigr) \bigl(1+x^{2} \bigr) \varOmega(f;\hat{\delta} )\frac {n^{2}}{e_{\upsilon}(nx)}\sum_{r=0}^{\infty} \frac{(nx)^{r}}{\gamma _{\upsilon}(r)} \\ \qquad{} \times \int_{0}^{\infty}\frac {e^{-nt}n^{r+2\upsilon\theta_{r} -1}t^{r+2\upsilon\theta_{r} }}{\gamma _{\upsilon}(r)} \biggl( 1+ \frac{1}{\hat{\delta} } \vert t-x \vert \biggr) \bigl( 1+(t-x)^{2} \bigr) d_{q}(t) \\ \quad=2 \bigl(1+\hat{\delta} ^{2} \bigr) \bigl(1+x^{2} \bigr) \bar{\varOmega} (f;\hat{\delta}) \frac{n^{2}}{e_{\upsilon}(nx)}\sum _{r=0}^{\infty} \frac{(nx)^{r}}{\gamma _{\upsilon}(r)} \int_{0}^{\infty}\frac{e^{-nt}n^{r+2\upsilon\theta_{r} -1}t^{r+2\upsilon\theta_{r} }}{\gamma_{\upsilon}(r)} \bigl\vert f(t) - f(x) \bigr\vert \,\mathrm{d}t \\ \qquad{}\times \biggl(1+(t-x)^{2}+\frac{1}{\hat{\delta} } \vert t-x \vert + \frac{1}{\hat{\delta} } \vert t-x \vert (t-x)^{2} \biggr) \\ \quad\leq2 \bigl(1+\hat{\delta}^{2} \bigr) \bigl(1+x^{2} \bigr) \bar{\varOmega}(f;\hat{\delta} ) \\ \qquad{}\times \biggl( 1+\mathcal{P}_{n,\upsilon}^{\ast}( \eta_{2};x)+\frac{1}{\hat {\delta} }\sqrt{\mathcal{P}_{n,\upsilon}^{\ast}(\eta_{2};x)}+ \frac{1}{\hat{\delta} }\sqrt {\mathcal{P}_{n,\upsilon}^{\ast}( \eta_{2};x)\mathcal{P}_{n,\upsilon}^{\ast}( \eta_{4};x)} \biggr).\end{gathered} $$

From Lemma 2.2, we easily see that

$$\begin{aligned} \mathcal{P}_{n,\upsilon}^{\ast}(\eta_{2};x) &\leq \mathcal{M}_{1,\upsilon} O{ \bigl( \mathfrak{W}_{\upsilon}(n) \bigr)} \bigl(1+x^{2} \bigr)\\ &\leq \mathcal{M}_{2,\upsilon}\bigl(1+x^{2} \bigr), \end{aligned} $$

where the constants $\mathcal{M}_{1,\upsilon}> 0$, $\mathcal{M}_{2,\upsilon}> 0$ and $\mathfrak{W}_{\upsilon}(n)=\max {\{}\frac{2}{n^{2}}, \frac {2}{n} (1 + \upsilon\frac{e_{\upsilon}(-nx)}{e_{\upsilon}(nx)} ) {\}}$.

And for the constants $\mathcal{M}_{3,\upsilon}>0 $ and $\mathcal{M}_{4,\upsilon}>0$, we have

$$\mathcal{P}_{n,\upsilon}^{\ast}(\eta_{4};x) \leq \mathcal{M}_{3,\upsilon} \bigl(1+x+x^{2}+x^{3} \bigr)\leq \mathcal{M}_{4,\upsilon}. $$

If we choose $\hat{\delta}=\sqrt{\mathfrak{W}_{\upsilon}(n)}$, $\mathcal{M}_{\upsilon}=1+\mathcal{M}_{2,\upsilon}+\mathcal{M}_{1,\upsilon}\mathcal {M}_{4,\upsilon}$, which easily leads to the result asserted by Theorem 6.2. □

## Conclusion

The present research article has an ample experience in applying appropriate properties to obtain uniform approximation results and an assessment of research methodologies to the approximation process. We establish a generalized version of the classic Phillips operators [24] by a Dunkl type generalization to the continuous functions connected with an extended exponential function. The point should be noted that in case of $\upsilon=0$, the operators (2.1) reduce to the classical Phillips operators given by [24]. The approximation obtained by these operators designed by Dunkl type provides a better generalization and an educational platform to the researcher to obtain the error estimations of the uniform convergence depending on *υ*.
